# Biofilm Removal and Bacterial Re-Colonization Inhibition of a Novel Erythritol/Chlorhexidine Air-Polishing Powder on Titanium Disks

**DOI:** 10.3390/ma11091510

**Published:** 2018-08-23

**Authors:** Magda Mensi, Andrea Cochis, Annamaria Sordillo, Francesca Uberti, Lia Rimondini

**Affiliations:** 1 Department of Surgery, Radiology and Public Health, Section of Periodontics, Università degli Studi di Brescia, 25121 Brescia (BS), Italy; magda.mensi@unibs.it (M.M.); annamaria.sordillo@gmail.com (A.S.); 2 Department of Health Sciences, Università del Piemonte Orientale UPO, 28100 Novara (NO), Italy; andrea.cochis@med.uniupo.it (A.C.); francesca.uberti@med.uniupo.it (F.U.)

**Keywords:** air-polishing, titanium, erythritol, chlorhexidine, biofilm, implants

## Abstract

Air-polishing with low abrasiveness powders is fast arising as a valid and mini-invasive instrument for the management of biofilm colonizing dental implants. In general, the reported advantage is the efficient removal of plaque with respect to the titanium integrity. In the present study, we evaluated the in situ plaque removal and the preventive efficacy in forestalling further infection of an innovative erythritol/chlorhexidine air-polishing powder and compared it with sodium bicarbonate. Accordingly, two peri-implantitis-linked biofilm formers, strains *Staphylococcus aureus* and *Aggregatibacter actinomycetemcomitans*, were selected and used to infect titanium disks before and after the air-polishing treatment to test its ability in biofilm removal and re-colonization inhibition, respectively. Biofilm cell numbers and viability were assayed by colony-forming unit (CFU) count and metabolic-colorimetric (2,3-Bis-(2-Methoxy-4-Nitro-5-Sulfophenyl)-2*H*-Tetrazolium-5-Carboxanilide) (XTT) assay. Results demonstrated that air-polishing performed with either sodium bicarbonate or erythritol/chlorhexidine was effective in reducing bacteria biofilm viability and number on pre-infected specimens, thus showing a similar ability in counteracting existing infection in situ; on the other hand, when air-polished pre-treated disks were infected, only erythritol/chlorhexidine powder showed higher post-treatment biofilm re-growth inhibition. Finally, surface analysis via mechanical profilometry failed to show an increase in titanium roughness, regardless of the powder selected, thus excluding any possible surface damage due to the use of either sodium bicarbonate or erythritol/chlorhexidine.

## 1. Introduction

Dental implants’ placement makes them part of the intra-oral microenvironment, where a complex microbial community tends to adhere to any available surface and build a biofilm [1,2,3].

Bacterial colonisation at implant surface occurs within 30 min of the implant trans-mucosal portion being connected to the surgical site, while a mature sub-gingival microbiota can be observed within a week [2,4]. Periodontal pathogens (*P. gingivalis*, *A. actinomycetemcomitans*, *T. forsythia*, *T. denticola*, *P. intermedia*) can be detected early, along with peculiar implant-colonising bacteria such as *E. coli* and *S. aureus* [2,4,5]. Shifts in the microbial load or in the relative amount of pathogens can lead to the development of peri-implant mucositis and peri-implantitis [3,6,7]. A regular and accurate disruption of the biofilm at the implant surface is therefore mandatory to prevent and treat peri-implant inflammatory diseases. Nowadays, there is no irrefutable evidence of the superiority of any mechanical or chemical biofilm control means over the others [8,9]. Although mechanical debridement with manual and/or power-driven instruments can lead to resolution of the inflammatory process [9], it may fail to restore the biocompatibility of the implant due to surface alteration and deposit of debris from the instruments, thus impairing cell viability and attachment [10,11]. For these reasons, a large literature is also focused on the development of innovative intrinsic antibacterial dental restorative compounds; so, dental materials can be directly doped with antibacterial agents such as metal ions [12,13], natural compounds such as polyphenols [14] or chemicals such as quaternary ammonium compounds [15,16] or peptides [17], as well as coupled with fillers containing the same antibacterial compounds [18].

Finally, it must also be considered that the increased surface roughness can lead to higher biofilm adhesion and accumulation [19].

Coming back to in situ biofilm removal techniques, air-polishing seems to constitute a valid tool for the supra- and sub-gingival management of biofilm at teeth and implants [20,21,22]. In-vitro application of air-polishing on micro-structured titanium surfaces and in simulated peri-implant defects seems to achieve a more successful biofilm removal when compared with various mechanical instrumentation means (e.g., plastic curettes and ultrasonic tips with chlorhexidine irrigation, the Vector system) and lasers (Er:YAG and Er,Cr:YSGG) [23,24,25,26,27]. Air-polishing also grants higher osteoblast viability on titanium surface compared with hand and ultrasonic instrumentation [23,27] and Er:YAG laser [25]. However, powders with a different composition or from different manufacturers can exploit peculiar effects on the treated surface [23,28,29]. Sodium bicarbonate is useful in biofilm removal from micro-structured titanium discs but alters the surface morphology [23,30], while glycine powder grants the same efficacy with minimal damage to the titanium, thanks its low abrasiveness [11,23,29,30]. Also, glycine seems able to reduce the bacterial re-colonisation of the treated surface [30,31].

Erythritol is a biocompatible [32,33], non-cariogenic [34], non-toxic sugar-alcohol [33] recently introduced in the formulation of a low-abrasiveness powder, in combination with chlorhexidine (erythritol/CHX). Among the polyols family, erythritol shows the highest inhibitory activity towards cariogenic bacteria [35] and *P. gingivalis* [36], both in-vitro and in-vivo. It can also decrease the adherence ability of several oral streptococci [37]. Air-polishing with erythritol/CHX in periodontal maintenance therapy showed comparable clinical outcomes to ultrasonic and manual debridement [21,22] and a higher decrease of sites positive for *A. actinomycetemcomitans* [21].

Currently, comparative studies into the efficacy of air-polishing with erythritol/CHX in titanium decontamination are still limited. Recent in vitro studies have shown that erythritol/CHX causes no changes in the topography of the implant neck [11] and exploits an inhibitory and microbicide activity against bacteria previously cultivated on sandblasted titanium discs [31,38], with a stronger anti-biofilm activity than that obtained by glycine [31].

The present in vitro study aimed to test the efficacy of air-polishing with an erythritol/CHX powder (AIR-FLOW^®^ PLUS, EMS Electro Medical Systems, Nyon, Switzerland) in biofilm removal and prevention of bacterial re-growth onto titanium disks; surface roughness changes were evaluated by profilometer. The antibacterial activity was assayed towards *S. aureus* and *A. actinomycetemcomitans* strains, two common peri-implant biofilm formers. Sodium bicarbonate powder served as a comparison.

## 2. Materials and Methods

### 2.1. Materials

For the experiments, 1 cm diameter, 2 mm thickness titanium grade II was used. Air-polishing treatments were performed using an AIR-FLOW^®^ HANDY 3.0 PERIO system (EMS Electro Medical Systems, Nyon, Switzerland) equipped with two different powders: (i) a sodium bicarbonate-based powder (AIR-FLOW^®^ CLASSIC COMFORT, EMS Electro Medical Systems, Nyon, Switzerland), and (ii) an erythritol/CHX powder (AIR-FLOW^®^ PLUS, EMS Electro Medical Systems, Nyon, Switzerland) with a ~14 µm granulometry and 0.3% chlorhexidine content. All other reagents were purchased from Sigma (Sigma-Aldrich, Milan, Italy).

### 2.2. Profilometry

Powders’ abrasiveness was evaluated by profilometry via mechanical profilometer (Surtronic 3+, Taylor-Hobson, Leicester, UK). Each titanium disk surface roughness was first evaluated prior to undergoing air-polishing treatment to set-up single-disk basic parameters. Air-polishing treatment was then performed on the previously analysed disks using one of the selected powders following the manufacturer’s instructed set-up to optimize the treatment as follow: 30 s length, maintaining 5 mm of distance and a 35° angle towards the surface. After the air-polishing phase, disk roughness was again evaluated by profilometer to verify any surface change due to powders abrasiveness.

### 2.3. Strains and Growth Conditions

Verified strains of two strong commercial biofilm formers—*Staphylococcus aureus* e *Aggregatibacter actinomycetemcomitans*—were purchased from the Leibniz Institute German Collection of Microorganisms and Cell Cultures (DSMZ, Braunschweig, Germany). Lyophilized bacteria were equilibrated following the manufacturer’s instructions into 1 mL of fresh Luria-Bertani (LB) broth for 20 min at room temperature; then, 100 μL of the bacteria solution was plated into LB agar plates and incubated at 37 °C for 24 h until single round colonies were formed. Fresh broth culture was prepared prior to each experiment by dissolving 3–4 single colonies into fresh LB medium until a 0.01 optical density (corresponding to 1 × 10^7^ cells/mL) was obtained by spectrophotometer analysis at 600 nm wavelength.

### 2.4. Preventive Anti-Adhesion Activity

To verify the ability of the test powders to prevent bacteria adhesion, titanium disks were pre-treated with the air-polishing technique prior to being infected. Autoclaved sterile disks were treated with either sodium bicarbonate powder or erythritol/CHX powder for 30 s, keeping 5 mm of distance and a 35° angle towards the surface, and then seeded onto 24-well plates and immediately submerged with 1 mL of fresh bacteria culture containing 1 × 10^7^ cells/mL (prepared as described in Section 2.3). The plate was incubated for 24 h at 37 °C and then bacteria number and viability were evaluated based on colony-forming unit (CFU) count and the metabolic-colorimetric (2,3-Bis-(2-Methoxy-4-Nitro-5-Sulfophenyl)-2*H*-Tetrazolium-5-Carboxanilide) (XTT) assay.

For the CFU count, the disks were moved to a new 24-well plate and washed 3 times with sterile PBS to remove non-adherent cells; biofilm was detached from disks surface by sonication and vortex (30 s each), collected and used to perform 6 ten-folder dilutions by mixing 20 μL of the bacteria suspension with 180 μL of sterile PBS. From each dilution, 20 μL were collected and spotted onto LB agar plates and incubated for 24 h at 37 °C; the day after, the number of viable colonies was counted as follows [39,40]:

CFU = [(number of colonies × dilution factor)^^(serial dilution)^]

where: number of colonies = countable single round colonies; dilution factor = dilution made from the initial 1 mL suspension; serial dilution = 1–6 ten-fold dilution areas where colonies were counted.

For the XTT analysis, the disks were moved to a new 24-well plate and washed 3 times with sterile PBS to remove non-adherent cells; then, each disk was submerged with 1 mL of XTT solution (1 mg/mL in PBS) and the plate was incubated 4 h in the dark. Finally, 100 μL from each disk supernatant was transferred to a new 96-well plate and, to evaluate optical density, evaluated by spectrophotometer (Victor, PerkinElmer, Waltham, MA, USA) at 570 nm wavelength. Untreated disks were used as a control and considered to have 100% viability; accordingly, test specimens’ results were normalized towards control and expressed as a% of it [32].

### 2.5. Plaque Removal

To test air-polishing decontamination ability, autoclaved sterile disks were infected as described in 2.4 for 24 h. Then, the air-polishing procedure was applied for 30 s, keeping 5 mm of distance and a 35° angle towards the surface. After air-polishing treatment, disks were stored for 20 min in the incubator at 37 °C, and then cell number and viability were assayed by CFU count and XTT evaluation as described previously in Section 2.4.

### 2.6. Morphological Analysis

To determine bacterial viability, a live/dead BacLight bacterial viability kit (Molecular Probes, Life Technologies Italia, Monza, Italy) was used. Experiments were performed following the manufacturer’s instructions; briefly, the working solution was prepared by mixing 3 μL/mL of both SYTO^®^9 and propidium iodide (both included in the BacLight bacterial viability kit from Molecular Probes, Life Technologies Italia, Monza, Italy) in sterile saline solution (0.9% NaCl). Then, 100 μL of working solution was gently spotted directly onto the specimen’s surface without any fixation step. Specimens were incubated at room temperature for 15 min in the dark, and then stained biofilms were examined by fluorescent microscopy (Leica 6500, Leica Microsystems, Basel, Switzerland).

### 2.7. Statistical Analysis of Data

Each experiment was performed using at least 3 specimens; obtained data were analysed by the SPSS software (IBM, Chicago, IL, USA) using the *ONE-way* ANOVA test followed by the Sheffè *post-hoc* analysis. Significance level was set at *p* < 0.05.

## 3. Results

### 3.1. Surface Analysis by Profilometry

**Figure 1:**
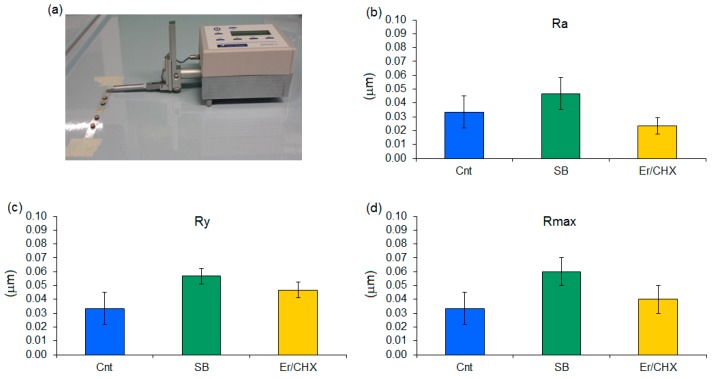
Profilometric surface roughness analysis of air-polishing-treated titanium disks (**a**). No significant differences were noticed between untreated control (Cnt) specimens and the test sodium bicarbonate (SB) and erythritol/CHX (Er/CHX) ones (*p* > 0.05) for all the surface Ra (**b**), Ry (**c**) and Rmax (**d**). Bars represent means and standard deviations.

Profilometry analysis results are reported in Figure 1a,d. After the air-polishing treatment, no statistically significant differences were noticed between the untreated specimens (considered as control) and the sodium bicarbonate and erythritol/CHX test specimens by profilometer analysis (schematized in Figure 1a). None of the considered values Ra (Figure 1b), Ry (Figure 1c) and Rmax (Figure 1d) reported significant differences in comparison with the untreated control. Thus, the use of neither sodium bicarbonate nor erythritol/CHX powders following the manufacturer’s suggested set-up of 30 s length, 5 mm of distance and a 35° angle towards the surface caused any significant modifications to the specimens’ surface roughness (*p* > 0.05).

### 3.2. Preventive Anti-Bacteria Adhesion Activity Evaluation

**Figure 2:**
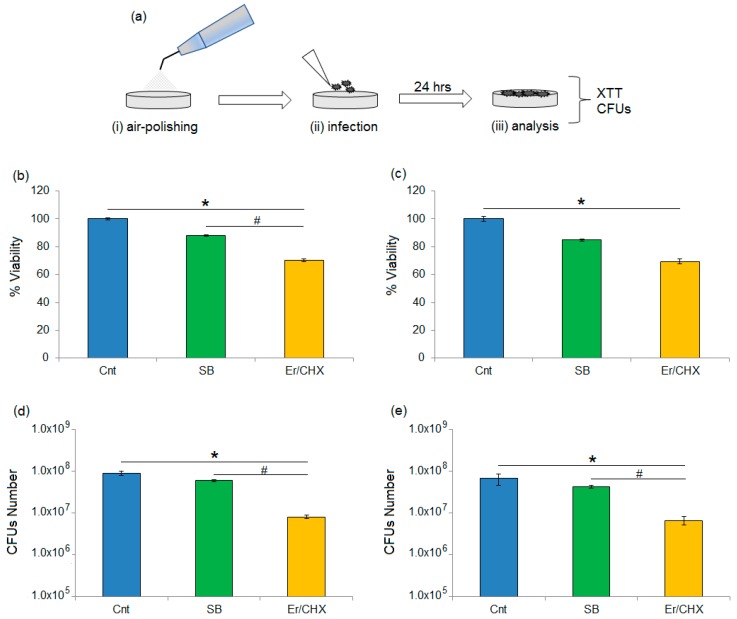
Preventive anti-bacteria adhesion ability evaluation. The application of erythritol/CHX (Er/CHX) powder by air-polishing procedure prior to the infection step (as schematized in (**a**)) was able to reduce *S. aureus* viability (**b**) and number (**d**) in a significant way in comparison with untreated controls (Cnt, *p* < 0.05, indicated by the *) and sodium bicarbonate (SB, *p* < 0.05, indicated by the #). Similarly, significant differences were noticed in *A. actinomycetemcomitans* viability (**c**) between erythritol/CHX and untreated controls (*p* < 0.05, indicated by the *) and between erythritol/CHX and untreated control (*p* < 0.05, indicated by the *) or sodium bicarbonate (*p* < 0.05, indicated by the #) when the CFU count was applied (**e**). Bars represent means and standard deviations.

The preventive use of the sodium bicarbonate and erythritol/CHX powders by means of the air-polishing procedure was efficient in prevention of bacteria adhesion. Results are reported in Figure 2a,e.

Air polishing was applied prior to specimens’ undergoing infection, as schematized in Figure 2a. Regarding *S. aureus* biofilm viability, significant differences were noticed between the untreated controls and erythritol/CHX-treated specimens (Figure 2b, *p* < 0.05, indicated by the *); moreover, significant differences were also noticed by comparing sodium bicarbonate and erythritol/CHX specimens (Figure 2b, *p* < 0.05, indicated by the #).

Considering *A. actinomycetemcomitans* viability, significant differences were noticed only by comparing the untreated and the erythritol/CHX-treated specimens (Figure 2c, *p* < 0.05, indicated by the *). No significant differences were noticed between untreated controls and the sodium bicarbonate between either of the two test powders (*p* > 0.05).

The XTT data seems to be confirmed by the CFU count (Figure 2d,e). In fact, the number of *S. aureus* colonies was significantly decreased by erythritol/CHX in comparison with untreated controls (Figure 2d, *p* < 0.05, indicated by the *) and sodium bicarbonate (Figure 2d, *p* < 0.05, indicated by the #).

A similar trend was confirmed for *A. actinomycetemcomitans* colony numbers, which mostly decreased with erythritol/CHX powder, with significant results in comparison to both untreated controls and sodium bicarbonate (Figure 2e, *p* < 0.05, indicated by the * and by the # respectively).

### 3.3. Plaque Removal Evaluation

**Figure 3:**
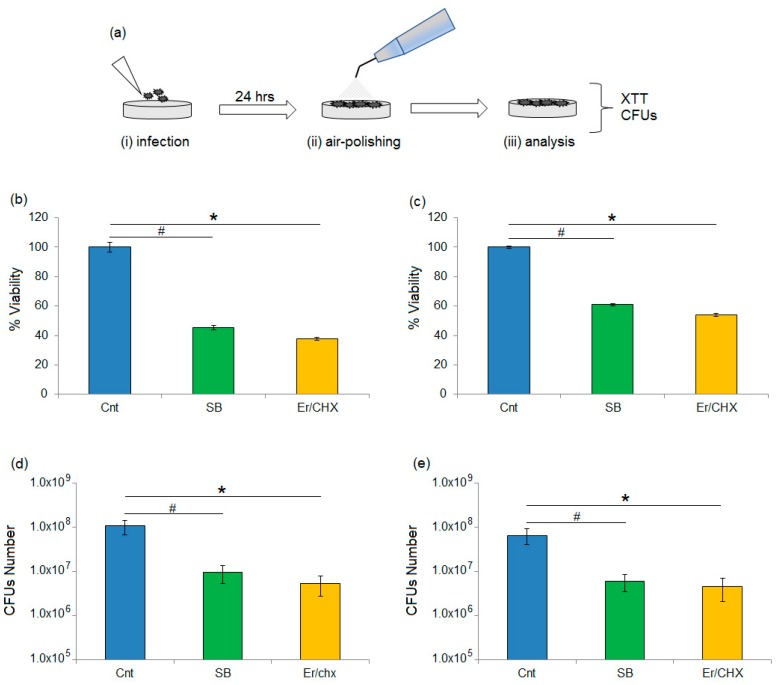
Plaque removal evaluation. When air polishing was applied onto pre-infected specimens (as schematized in (**a**)), it significantly reduced *S. aureus* (**b**) and *A. actinomycetemcomitans* (**c**) viability in comparison with the untreated controls (*p* < 0.05, indicated by the * and #, respectively). Likewise, bacteria number was also significantly reduced (*p* < 0.05) by sodium bicarbonate (SB) and erythritol/CHX (Er/CHX) powders in comparison with untreated controls (Cnt) when both *S. aureus* ((**d**), indicated by the * and #) and *A. actinomycetemcomitans* ((**e**), indicated by the * and #) were considered. No significant differences were observed between the two test powders (*p* > 0.05). Bars represent means and standard deviations.

The air-polishing technique performed with either sodium bicarbonate or erythritol/CHX was effective in reducing bacteria biofilm viability and number in pre-infected specimens. Results are reported in Figure 3a–e.

Air polishing was applied after specimens’ infection, as schematized in Figure 3a. Similar results were obtained considering *S. aureus* (Figure 3b–d) and *A. actinomycetemcomitans* (Figure 3c–e) biofilm; by XTT assay, it was demonstrated that bacteria viability was significantly (*p* < 0.05) reduced after air-polishing treatment. The viability of treated groups (sodium bicarbonate and erythritol/CHX) showed significant results in comparison with the untreated control in both *S. aureus* (Figure 3b, indicated by the *) and *A. actinomycetemcomitans* (Figure 3c, indicated by the #). No significant differences were noticed between the two test powders (*p* > 0.05).

Bacteria viability data were then confirmed by the CFU count (Figure 3d,e); in fact, the number of bacteria colonies was reduced in a significant manner (*p* < 0.05) by the appliance of air-polishing procedure for both *S. aureus* (Figure 3d, indicated by the *) and *A. actinomycetemcomitans* (Figure 3e, indicated by the #). As previously shown by the XTT analysis, CFU count also evidenced no significant differences between the sodium bicarbonate and the erythritol/CHX powders (*p* > 0.05).

### 3.4. Biofilm Morphology

**Figure 4:**
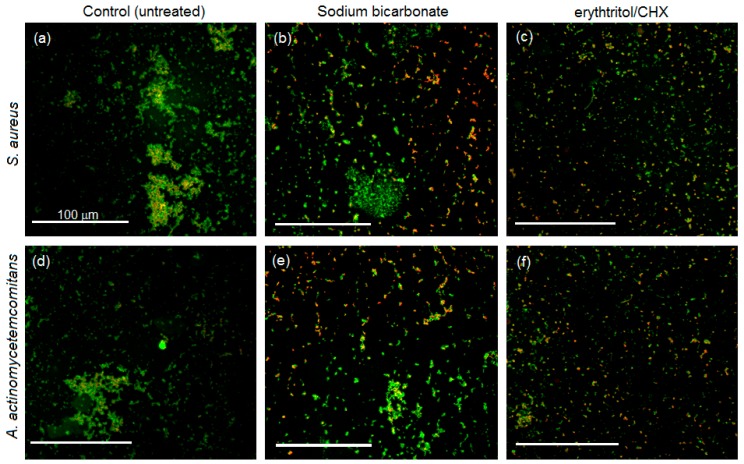
Live/Dead staining of specimens infected after air polishing pre-treatment. Strong biofilm formation was noticed only in control specimens for both *S. aureus* (SA, upper panel) and *A. actinomycetemcomitans* (AA, lower panel). In contrast, the use of air polishing decreased the formation of biofilm aggregates; in particular, erythtritol/CHX appliance determined the formation of mostly single colonies, thus preventing bacteria aggregation into biofilm. Magnification = 20×, bar scale = 100 μm. (**a**) SA, control; (**b**) SA, sodium bicarbonate; (**c**) SA, erythtritol/CHX; (**d**) AA, control; (**e**) AA, sodium bicarbonate; (**f**) AA, erythtritol/CHX.

**Figure 5:**
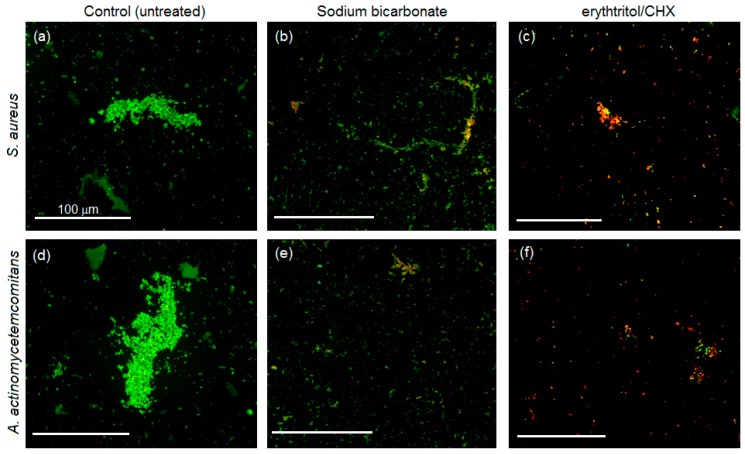
Live/Dead staining of specimens treated with polishing after 24 h infection. Control specimens showed a massive presence of biofilm aggregates for both *S. aureus* (SA, upper panel) and *A. actinomycetemcomitans* (AA, lower panel); on the other hand, air polishing appliance was effective in strongly reducing bacteria number and biofilm formation. Magnification = 20 ×, bar scale = 100 μm. (**a**) SA, control; (**b**) SA, sodium bicarbonate; (**c**) SA, erythtritol/CHX; (**d**) AA, control; (**e**) AA, sodium bicarbonate; (**f**) AA, erythtritol/CHX.

Live/Dead assay results are reported in Figure 4 and Figure 5, respectively.

In the air polishing preventive efficacy evaluation (Figure 4), as expected, evident biofilm formation was observed in the untreated controls for both *S. aureus* (upper panel) and *A. actinomycetemcomitans* (lower panel). Differently, looking at the air polished specimens, a general reduction in terms of bacteria number and biofilm formation was appreciable. In particular, for *S. aureus*, some differences were also noticed by comparing sodium bicarbonate and erythtritol/CHX, as only the former showed typical biofilm aggregates, whereas the latter displayed mostly single colonies.

Similarly, when air polishing was applied after infection (Figure 5), a marked difference was noticed when comparing untreated controls and test specimens. In fact, without any treatment, a massive biofilm formation was evident on the surface of titanium disks for both the tested *S. aureus* (upper panel) and *A. actinomyctemecomitans* (lower panel). On the other hand, by applying air polishing with both sodium bicarbonate or erythtritol/CHX powders, specimens’ surfaces were much less colonized; moreover, no evident biofilm aggregate accumulation was detected, thus demonstrating air polishing efficacy.

## 4. Discussion

The development of peri-implant inflammatory disease depends on the presence and growth of biofilm at the implant surface [41]. Air-polishing with low abrasiveness powder is stepping in as a valid and mini-invasive instrument for the management of dental implants biofilm as recently described by Schwarz et al. [20], who demonstrated that it can be successfully applied in mucositis cases as a mono-therapy or in combination with ultrasonic debridement.

In the present study, a new erythritol/CHX powder was chosen (AIR-FLOW^®^ PLUS, EMS Electro Medical Systems, Nyon, Switzerland) and compared to a sodium bicarbonate one in terms of biofilm removal efficiency, surface roughness alteration and preventive anti-biofilm activity.

To test the efficacy in biofilm removal, Grade II titanium disks were infected for 24 h with the periodontal *S. aureus* and *A. actinomycetemcomitans* pathogens prior to undergoing air-polishing treatment*.* Results showed that both erythritol/CHX and sodium bicarbonate powders were significantly effective in reducing the quantity and metabolic activity of bacterial cells**.** These findings are in line with the results of Drago et al. [31] who tested the bacterial viability of *S. aureus*, *C. albicans* and *B. fragilis* biofilm on titanium sandblasted disks after 5 s of air-polishing with glycine and erythritol/CHX. The treatment with erythritol/CHX powder obtained a significantly higher reduction of all bacterial strains, compared with both glycine powder and a mechanical control treatment.

These positive results could be related to the evidence that both erythritol and chlorhexidine are known to exert an anti-biofilm/antimicrobial activity. In particular, chlorhexidine has a well-known immediate and posterior antimicrobial effect thanks to its substantivity [42,43], while erythritol shows different mechanisms of action. Söderling et al. [37] demonstrated that erythritol decreases the polysaccharide-mediated bacterial adherence of different Streptococcus strains. Of periodontal relevance, Hashino et al. [36] showed that erythritol is able to suppress the growth of a *P. gingivalis*–*S. gordonii* biofilm, interfering with different metabolic pathways and inhibiting the nucleotide and matrix biosynthesis. Regular administration of erythritol has been advocated as a caries-prevention regimen: Mäkinen et al. [35] showed that a 6-month-long daily use of erythritol-containing chewable tablets and dentifrice is able to reduce the plaque and saliva levels of S. *mutans* streptococci, while Falony at al. [44] proved the caries-preventive effect of a daily dose of erythritol during a 3-year intervention, then observing persistence of the benefit up to 3 years after the end of the administration. However, there is currently no evidence of erythritol substantivity after a single administration or through its application as a single-component via air-polishing. 

Regarding the preventive anti-biofilm efficacy of the erythritol/CHX powder, Drago et al. [31] provided the first evidence of prolonged activity, analysing the recovery level of the residual biofilm after 16–18 h of incubation. In fact, the disks treated with erythritol/CHX showed around 50% less viable cells than the control specimens. In this work, comparable results were achieved as the percentage of metabolic active bacteria decreased of about 30% by the erythritol/CHX treatment.

The authors speculate that this outcome might be due to the substantivity of the CHX on the titanium disks surface.

The differences noticed between XTT and CFUs data can be explained by the Live/Dead assay; in fact, the reduction in terms of metabolic activity detected by XTT can be reasonable related by the strong reduction of the colonies number. Meanwhile, in the control specimens, the disk surface presented a massive biofilm accumulation, the preventive or in situ removal air polishing application determined a strong reduction of the biofilm community, leading to the accumulation of mostly single colonies.

Of major concern during plaque removal at the implant surface is possible surface alteration, since increased roughness is related to augmented plaque accumulation [45,46,47]. In the present study, a mechanical profilometer was used to evaluate the titanium disks roughness before and after the application of air-polishing with sodium bicarbonate or erythritol/CHX powder for 30 s. The profilometry failed to prove any statistically significant difference in surface roughness between treated and untreated specimens, regardless of the powder used, thus excluding the possibility to favour the bacterial adhesion. This finding does not mean that sodium bicarbonate and erythritol/CHX powders have an equal effect on titanium surface. Cochis et al. [30] analysed the morphological change induced by air-polishing on titanium disks through both laser profilometry and scanning electron microscopy (SEM). Even if the mean and maximum roughness measured by the profilometer did not reach a statistically significant difference after application sodium bicarbonate powder, the SEM observation revealed newly formed roughness and craters. Low abrasiveness powders such as glycine and erythritol/CHX seem to cause almost no observable morphological changes at SEM analysis [11,30]. However, Schmidt et al. [11] in an in-vitro study compared two different glycine powders and an erythritol/CHX by SEM images revealing that the erythritol/CHX powder results in the least surface modification.

The clinical significance of the surface alternation is uncertain and only biofilm viability tests performed after the application of air-polishing can give an insight. Cochis et al. [30] in the in-vivo part of their study exposed the treated titanium disks to the oral environment. The microbiological evaluation could not reveal any statistically significant difference in plaque accumulation between untreated and sodium bicarbonate-treated specimens, confirming no increase in bacterial retention. 

Even if the present study failed to prove any roughness and plaque accumulation increase after air-polishing with sodium bicarbonate, it is important to consider that the application lasted for 30 s, simulating a single session of prophylaxis. In the clinical reality, patients with implant-supported restorations should undergo a session of maintenance therapy every 5–6 months [48]. As a consequence, with a long-term application of sodium bicarbonate, a cumulative increase of surface alteration can be supposed and could become of clinical relevance. It is important to remember that any damage to the titanium may decrease fibroblast adhesion and, hence, implant biocompatibility [49]. Moreover, sodium bicarbonate powder is proven to cause damage to the gingival tissue [50], making it necessary to aim the air-polishing jet away from soft tissues and keep a distance from the gingival margin during the in-vivo application. 

The present study has some limitations. The first is related to the use of mono-bacteria biofilm model. Exposure of the titanium disks to the actual oral biofilm in its complexity would give a more realistic picture of the anti-biofilm activity of the erythritol/CHX powder. The second is related to the lack of definitively proved cytocompatibility of titanium because of chemical and physical-chemical modification after erythritol/CHX powder. Actually, even if erythritol was claimed to be a non-toxic molecule [51], CHX has a demonstrated detrimental effect on eukaryotic cells in-vitro viability [39]. Therefore, biocompatibility issue might be relevant in case of erythritol/CHX powder application for biofilm debridement in surgical treatment of periimplantitis.

## 5. Conclusions

Both sodium bicarbonate and erythritol/CHX powders are good tools for air-polishing at the implant surface. None of the powders determined a significant increase in titanium surface roughness, thus reducing the possibility to favour bacteria adhesion. Sodium bicarbonate and erythritol/CHX resulted effective in plaque removal and adhesion prevention, with a superior anti-biofilm effect towards the considered strains showed by the erythritol/CHX powder.
